# Clinical Features and Molecular Markers on Diffuse Midline Gliomas With H3K27M Mutations: A 43 Cases Retrospective Cohort Study

**DOI:** 10.3389/fonc.2020.602553

**Published:** 2021-02-15

**Authors:** Yuan Wang, Lan-lan Feng, Pei-gang Ji, Jing-hui Liu, Shao-chun Guo, Yu-long Zhai, Eric W. Sankey, Yue Wang, Yan-rong Xue, Na Wang, Miao Lou, Meng Xu, Min Chao, Guo-Dong Gao, Yan Qu, Li Gong, Liang Wang

**Affiliations:** ^1^ Department of Neurosurgery, Tangdu Hospital, Fourth Military Medical University, Xi’an, China; ^2^ Department of Pathology, Tangdu Hospital, Fourth Military Medical University, Xi’an, China; ^3^ Department of Neurosurgery, Duke University Hospital, Durham, NC, United States; ^4^ Department of Health Statistics, Fourth Military Medical University, Xi’an, China; ^5^ National Time Service Center, Chinese Academy of Sciences, Xi’an, China; ^6^ School of Optoelectronics, University of Chinese Academy of Sciences, Beijing, China

**Keywords:** diffuse midline glioma, H3K27M, radiotherapy, P53, KPS

## Abstract

**Purpose:**

Diffuse midline gliomas (DMG) with H3K27M mutations have been identified as a rare distinctive entity with unique genetic features, varied molecular alterations, and poor prognosis. The current study aimed to evaluate the clinical characteristics and profile of molecular markers on patients with a DMG harboring H3K27M mutations, and explore the impact of this genetic makeup on overall survival.

**Methods:**

We retrospectively analyzed 43 consecutive patients diagnosed with a DMG harboring H3K27M mutations (age range 3 to 75 years) and treated in a tertiary institution within China between January 2017 to December 2019. Various clinical and molecular factors were evaluated to assess their prognostic value in this unique patient cohort.

**Results:**

The median overall survival (OS) was 12.83 months. Preoperative Karnofsky Performance Score (KPS) and adjuvant radiotherapy were found to be independent clinical parameters influencing the OS by multivariate analysis (*p* = 0.027 and *p* < 0.001 respectively). Whereas extent of tumor resection failed to demonstrate statistical significance. For molecular markers, P53 overexpression was identified as a negative prognostic factor for overall survival by multivariate analysis (*p* = 0.030).

**Conclusion:**

Low preoperative KPS, absence of radiotherapy and P53 overexpression were identified as predictors of a dismal overall survival in patients with DMG and H3K27M mutations.

## Introduction

Diffuse midline glioma (DMG) with histone H3K27M mutation is a novel entity that was added to the 2016 World Health Organization (WHO) classification of tumors of the Central Nervous System (CNS), on the principle of integrated diagnosis with combinations of characteristics from both histological and molecular aspects (1, 2). This distinct entity is an aggressive tumor that corresponds to grade IV glioma, regardless of their histological grade, and is described as an infiltrative glial neoplasm with predominately astrocytic differentiation and a K27M mutation in the histone H3 gene (either H3F3A or HIST1H3B/C) (1, 3).

In accordance to the diagnostic criteria outlined by the WHO, DMG with H3K27M mutations have a diffuse growth pattern and are restricted to the midline structures, such as the thalamus, brainstem, cerebellum and spinal cord (4). These tumors are most frequently observed in the pediatric population, but adults are also commonly affected (5). As an aggressive malignant tumor, prognosis is considerably poor, and the 2-year survival rate is less than 10% for patients receiving standard treatment including surgery and adjuvant chemoradiation (6).

Given the rarity and novelty of DMG with H3K27M mutation in adults (7), several studies have sought to describe the incidence and clinical behavior of these tumors (8, 9). However, not all reported cases adequately fulfill the diagnostic criteria for DMG with H3K27M mutation. For example, non-midline gliomas with H3K27M mutation or circumscribed H3K27M-mutant cases have also been included in prior studies (6, 10). Owing to the unique deep-seated location of DMG, surgical treatment is often challenging and might lead to suboptimal resection or even failure to obtain an adequate biopsy specimen. As such, limited surgical outcomes data has been reported and further investigation of relevant molecular markers and associated genetic alterations is still needed.

In the current study, we retrospectively explored the clinical and molecular characteristics of 43 patients who were diagnosed with a DMG with H3K27M mutation between January 2017 and December 2019 at our institution. Our findings highlight some of the factors that influence patient survival and the potential cooperative genetic alterations for patients with DMG with H3K27M mutations.

## Materials and Methods

### Patient cohort and Identification

This retrospective study was reviewed and approved by our Institutional Review Board (IRB). The relevant demographic and clinical data on all glioma cases were obtained and identified from the institutional electronic medical record (EMR) system. For each case, the diagnosis and pathological classification were confirmed according to the 2016 WHO classification of CNS tumors. Consecutive cases of diffuse midline gliomas were selected from January 2017 to December 2019, as H3K27M immunohistochemistry (IHC) began to be regularly performed on glioma patients in our hospital starting in January 2017. Pre-operative Magnetic resonance imaging (MRI) was reviewed by two neurosurgeons (P-G Ji and J-H Liu) to determine the location of the gliomas. The primary tumor location was reported and categorized as thalamus, midbrain, pons, medulla oblongata, basal ganglia, spinal cord or cerebellum. Patients were followed under the standard treatment protocol for all CNS tumor patients at our institution and the clinical status of all patients at last follow-up was recorded.

For evaluation of the extent of resection, gross total resection (GTR) was defined as no residual tumor remnant (100%) seen with postoperative MRI imaging. Subtotal resection (STR) was defined as less than 90% extent of resection, while the partial resection (PR) was defined as less than 50% resection of the tumor. Postoperative computed tomography (CT) and MRI were applied within 48 h postoperatively.

In total, 43 cases of diffuse midline gliomas with H3K27M mutation were included for analysis.

### Immunohistochemistry and Pathology Review

Tissue blocks embedded with formalin-fixed paraffin (FFPE) and hematoxylin and eosin (H&E)–stained slides were retrieved from the Department of Pathology. Five to eight unstained slides with 4-μm-thick sections were prepared for further IHC evaluation for each case. IHC staining was performed for the differential diagnosis. IHC analysis included H3K27M, trimethylation of Lys27 (H3K27Me3), O-6-methylguanine DNA methyltransferase (MGMT), IDH-1, ATRX, p53, fibrillary acidic protein, Oligo2, nestin, NeuN, Syn, S-100, and Ki-67. IHC was interpreted by a neuro-pathologist and stains were registered as positive, negative or uninterpretable.

### DNA Extraction and Mutation Detection

Sanger sequencing (SINOMD, Beijing, China) was used to determine the frequency of mutations in IDH1 and IDH2. Sanger sequencing were performed using an ABI‐3130 DNA Analyzer (Applied Biosystems).

Methylation-specific quantitative PCR (qMSP) was applied to detect the status of MGMT promoter methylation. Genomic DNA was extracted from paraffin section and converted by bisulfite. The QIAamp DNA FFPE tissue Kit (Tiangen Biotech, Beijing, China) and DNA Bisulfite Conversion Kit (Tiangen Biotech, Beijing, China) were used according to the manufacturer’s instructions. The converted DNA was mixed with MGMT methylation mixture (SINOMD, Beijing, China). Real-time quantitative polymerase chain reaction was performed by ABI 7500 Fast Dx (Applied Biosystems Co. Ltd., US) at 95°C for 3 min, then 45 cycles of 95°C for 15 s and 60°C for 45 s.

### Statistics

Continuous variables were reported by mean and standard deviation or median and interquartile range (IQR) and analyzed using the unpaired Student *t* test or Wilcoxon Sum Rank test, while H3K27M mutations and related categorical variables were summarized using Pearson Chi-square or Fischer’s exact test. Kaplan-Meier curves and log-rank test were used to compare survival between patients with or without changes. In this study, GraphPad Prism (Version 7.10, La Jolla, California, USA) and SPSS statistics (version 22.0, IBM corp., Armonk, New York, USA) were applied for the statistical analysis.

## Results

In our present study, 917 cases of histologically confirmed glioma (January 2017 to December 2019) were retrieved from the Tangdu Hospital Medical Record System, including circumscribed and diffused gliomas. By retrieving the data, the incidence of pediatric (<21 years old) glioma is 131/917 (14.3%), and incidence of adult (≥21 years old) glioma is 786/917 (85.7%). Of these, 43 cases were diagnosed as DMG with H3K27M mutation (n = 43/917, 4.69%). According to the diagnostic criteria of diffuse midline gliomas with H3K27M mutant, WHO IV, all 43 cases were retrospectively evaluated.

### Demographic Features of DMG Patients With H3K27M Mutations

Of the 43 cases confirmed as DMG with H3K27M mutant, WHO IV, 26 (60.5%) were males and 17 (39.5%) were females. At the time of diagnosis, the patient’s age ranged from 3 to 75 years, with the median age of 38 (IQR: 17-50). In our cohort, most of the patients with DMG were adults (aged≥21 years old, 30/43, 69.77%), with 13 (30.23%) children (<21 years old) included in the present study.

In our study, the majority of tumors were located in the thalamus (33/43, 76.74%), followed by the brainstem (midbrain [10/43, 23.26%], pons [5/43, 11.63%], and medulla [4/43, 9.30%]). Since in some cases tumors are multifocal, the sum of the tumor location is greater than the total case number. Preoperative Karnofsky performance status (KPS) scores ranged from 20 to 90 (median 80). Limb weakness or numbness was most frequently observed (n = 25, 58.14%) as a presenting symptom. Other symptoms included headache (n = 12, 27.91%), hydrocephalus with nausea/vomiting symptoms (n = 12, 27.91%), visual disturbances (n = 6, 13.95%), epilepsy (n = 3, 6.98%), dizziness (n = 8, 18.60%), and memory loss (n = 2, 4.65%). Tumors were identified incidentally in 2 (4.65%) asymptomatic patients. One case was unintentionally detected after a minor traffic accident, and the other case was found by routine physical examination with a head CT scan.

The baseline demographic and clinical features of these cases are summarized in **Tables 1** and **2**.

**Table 1:** Demographic features of 43 patients of diffused midline glioma with H3K27M mutant.

Case ID	Gender	Age (years)	Locations		Surgery	Pre-op KPS	Treatment	OS (Months)	Status
1	Male	10	Left	Th	PR	30	RT+TMZ	41.87	Survive
2	Female	11	Right	Th+LV	GTR	80	RT+TMZ	5.83	Dead
3	Male	48	Left	Th+Mid	PR	60	None	1.33	Dead
4	Male	25	Right	Th+CP+Pon	PR	70	None	1.53	Dead
5	Female	9	Right	Th+BG	GTR	90	RT+TMZ	22.90	Survive
6	Male	63	Right	Th+BG	STR	70	NA	16.23	Dead
7	Male	9	Right	Pon+Med	PR	90	RT	17.70	Dead
8	Female	3	Left	Th+BG	GTR	80	RT	21.43	Survive
9	Female	41	Right	Th	STR	90	RT+TMZ	13.53	Survive
10	Male	50	Midline	Med+Cev	Biopsy	90	RT+TMZ	19.87	Survive
11	Female	26	Right	Th	PR	90	NA	15.50	Survive
12	Male	44	Right	Th+Cca	GTR	80	NA	0.33	Dead
13	Male	56	Right	Th+Cca	STR	60	NA	2.10	Dead
14	Male	5	Midline	Mid	PR	80	Herbal	10.03	Survive
15	Female	53	Midline	Th+Mid+Cere	Biopsy	30	None	2.80	Dead
16	Male	75	Right	Th+Cca	GTR	80	None	0.03	Dead
17	Male	71	Right	Th	STR	90	RT+TMZ	8.93	Survive
18	Male	40	Left	Th	STR	60	RT+TMZ	8.03	Survive
19	Male	14	Midline	Pon	STR	70	RT+TMZ	7.73	Survive
20	Male	30	Right	Med	Biopsy	90	RT+TMZ	7.57	Survive
21	Male	17	Left	Th	GTR	80	RT	4.80	Dead
22	Female	47	Midline	Th+Cca+Mid	STR	60	RT+TMZ	7.13	Dead
23	Male	59	Midline	V4	GTR	90	Herbal	7.33	Survive
24	Male	11	Right	Th+Cca+Pon	GTR	30	None	2.37	Dead
25	Male	33	Midline	Th+Mid	STR	90	RT	5.33	Survive
26	Male	29	Right	Th	STR	70	RT+TMZ	24.03	Survive
27	Female	43	Right	Th	PR	40	None	0.17	Dead
28	Female	19	Left	Th	PR	30	RT+TMZ	12.83	Dead
29	Female	27	Right	Th	PR	50	RT+TMZ	11.63	Dead
30	Male	50	Right	Th	PR	90	RT+TMZ	12.20	Dead
31	Male	55	Right	Th	STR	80	RT+TMZ	30.33	Survive
32	Female	13	Right	Th+LV+Mid	PR	30	RT+TMZ	7.53	Dead
33	Male	35	Left	Th	PR	90	TMZ	7.57	Dead
34	Female	59	Left	Th	PR	90	TMZ	8.20	Dead
35	Female	38	Right	Th	PR	60	RT+TMZ	25.03	Survive
36	Male	40	Left	Th	PR	70	TMZ	7.50	Dead
37	Female	17	Right	Th	PR	70	None	19.40	Survive
38	Female	63	Left	Th+CP	STR	80	None	4.87	Dead
39	Male	42	Left	Mid	PR	90	RT+TMZ	34.63	Survive
40	Female	14	Left	Th+Mid	PR	70	RT+TMZ	27.50	Survive
41	Female	33	Left	Mid	STR	90	RT+TMZ	25.97	Survive
42	Male	45	Left	Pon+Mid	Biopsy	20	None	1.50	Dead
43	Male	50	Midline	Med+Cere	PR	70	None	0.17	Dead

OS, overall survival; PFS, progression-free survival; Th, thalamic glioma; Mid, midbrain; GTR, gross total resection; RT, radiotherapy; TMZ, temozolomide; LV, lateral ventricle; STR, subtotal resection; V4, fourth ventricle; PR, partial resection; Cca, corpus callosum; BG, basal ganglia; CP, cerebral peduncle; Pon, pontine; Med, Medulla; Cev, cervical cord; Cere, cerebellum; KPS, Karnofsky performance status.

**Table 2:** Clinical features of 43 patients of diffused midline glioma with H3K27M mutant.

Characteristic	Cohort	*n*	%
Sex	Male	26	60.47
	Female	17	39.53
Tumor location*	Thalamus	33	76.74
	Later ventricle	2	4.65
	Cerebral peduncle	5	11.63
	Midbrain	10	23.26
	Pontine	5	11.63
	Basal ganglia	3	6.98
	Medulla	4	9.30
	Cervical cord	1	2.33
	Corpus callosum	5	11.63
	Cerebellum	2	4.65
	Fourth ventricle	1	2.33
Initial symptoms	Headache	12	27.91
	Limb weakness	25	58.14
	Dizziness	8	18.60
	Epilepsy	3	6.98
	Nausea/vomiting	12	27.91
	Memory loss	2	4.65
	Visual disturbances	6	13.95
	Incidental finding	2	4.65
Extent of resection	GTR	8	18.60
	STR	12	27.91
	PR	19	44.19
	Biopsy	4	9.30
Adjuvant therapy	None	14	32.56
	RT only	4	9.30
	RT+TMZ	20	46.51
	TMZ	3	6.98

*Since the tumor invades multiple brain regions, the sum of the tumor location is greater than the total of case number.

SD, standard deviation; GTR, gross total resection; STR, subtotal resection; PR, partial resection; PFS, progression-free survival; OS, overall survival; RT, radiotherapy; TMZ, temozolomide; KPS, Karnofsky performance status.

### Surgical Treatment and Follow-Up

PR and STR was performed in the majority of patients given the high risk of iatrogenic morbidity associated with GTR of thalamic and other midline lesions. For lesions located in the posterior fossa, including the brainstem and spine, surgical debulking or biopsy was considered as the primary surgical option. Ultimately, GTR was achieved in 8 patients (18.60%), STR was performed in 12 patients (27.91%), and PR was in 19 cases (44.19%), while 4 patients received stereotactic biopsy (9.30%). Representative images of extent of tumor resection are listed in **Supplementary Figure 1**.

All patients were followed postoperatively according to the standard protocol at our institution. Clinical status was recorded at last follow-up. Follow-up duration or time to death were recorded for all patients. In our cohort, there was no loss of follow-up and the median follow-up duration was 8.03 (0.03-41.87) months. Adjuvant chemoradiation was advised for all patients. However, only 20 patients (46.51%) received prescribed adjuvant both RT and temozolomide (TMZ)-based chemotherapy. Four patients (9.30%) completed RT only, and three patients (6.98%) received TMZ only. Two patients (4.65%) received other treatments, including traditional Chinese medicine and herbal therapy. Unfortunately, there were 14 patients (32.56%) who did not receive any additional treatment after surgery. At last follow-up, 20 (46.51%) patients were still alive, while 23 (53.49%) patients were deceased.

### IHC Features and Molecular Characteristics

Pathologic assessment including IHC was performed per institutional protocol for all tumor specimens. According to the 2016 WHO classification of CNS tumors, typical morphological features of glioma were presented in all cases. However, H3K27me3 and MGMT status was not available for every patient in this cohort with IHC.

**Figure 1:**
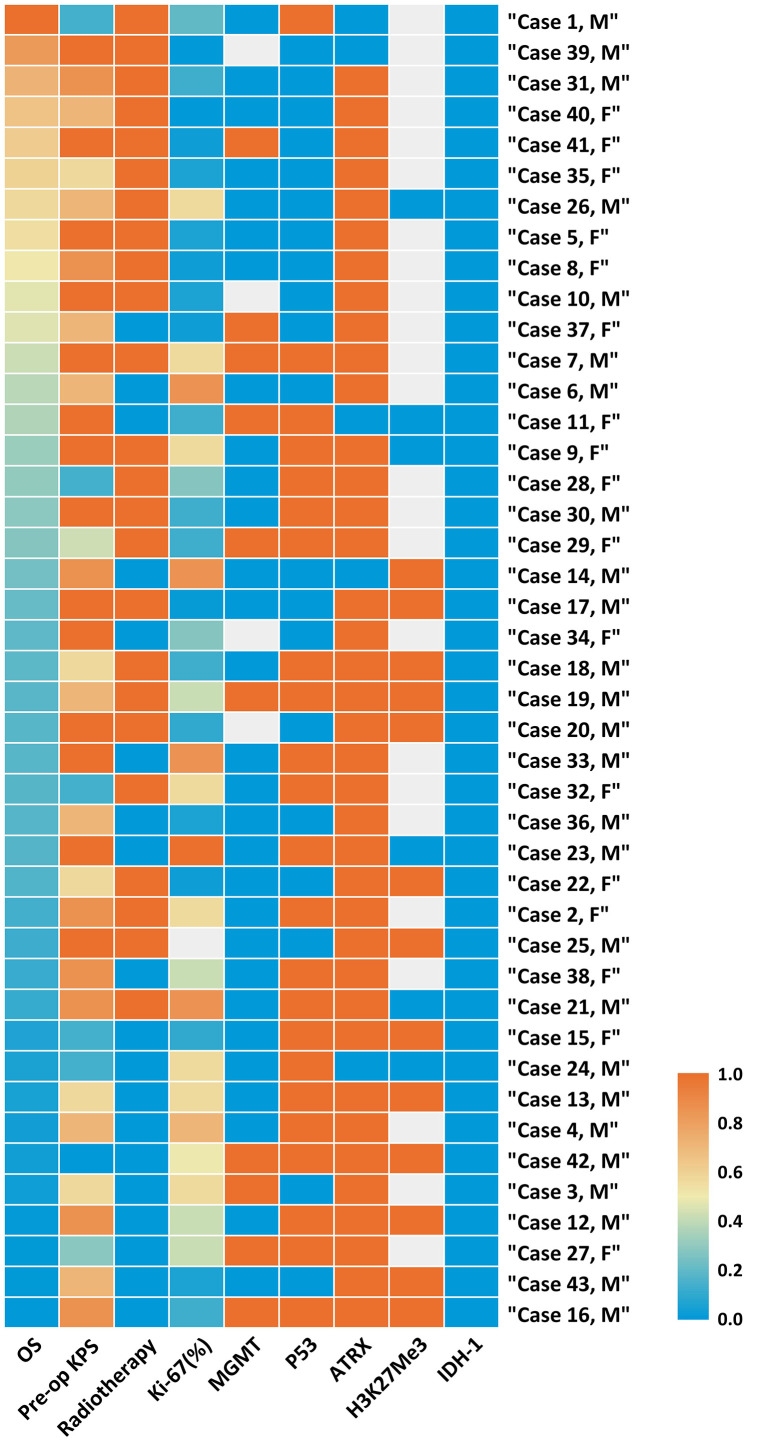
Heatmap of clinical and molecular features of 43 cases of Diffused Midline Glioma with H3K27M mutant. Each horizontal bar corresponds to a case and all aligned vertical bars across the variables correspond to the same case. The lowest value (0.0) in each column is blue, the highest value (1.0) in each column is red, while missing value is gray (see color scale). Different color densities represent the corresponding ratios. The first column order is arranged by OS decrement. Corresponding value in each cell could be referred in **Supplementary Table 1**.

In accordance with the 2016 WHO classification, all patients in our cohort presented with a H3K27M mutation, while the status of H3K27me3 expression was checked in 19 cases, in which 13 (68.42%) cases were found to have loss of expression, implying a decrease of H3K27Me3. p53 was overexpressed in 23 of 43 cases (53.49%), and loss of ATRX (loss of expression) was found in 38 of 43 cases (88.37%) (**Figure 1**, **Table 3**, and **Supplementary Table 1**).

**Table 3:** Univariate analysis on overall survival in patients of diffused midline glioma with H3K27M mutant.

Variables	Cohort	*n*	Median/mean OS(months)	95% CI	*p* value
Sex	Male	26	12.20	0.00–25.28	0.512
	Female	17	16.52	11.44–21.62	
Age (years)	≤21 years	13	25.30	15.70–34.91	0.282
	>21 years	30	11.63	5.18–10.08	
Extent of resection	GTR or STR	20	17.99	12.07–23.91	0.620
	PR or Biopsy	23	12.20	5.84–18.56	
Pre-op KPS	<70	13	7.13	1.07–13.19	0.027*
	≥70	30	21.06	15.62–26.49	
Adjuvant therapy	RT	24	29.79	23.11–36.47	<0.001*
	RT without	19	2.80	0.00–6.74	
Molecular markers	H3K27Me3 retention	6	17.22	9.48–24.95	0.332
	H3K27Me3 decrease	13	7.13	0.00–15.49	
	ATRX retention	5	33.97	20.12–47.82	0.171
	ATRX loss	38	12.20	5.89–18.51	
	P53 retention	20	25.62	19.59–31.65	0.002*
	P53 overexpression	23	7.53	3.52–11.54	
	*IDH1* (WT)	43	–	–	–
	*IDH2* (WT)	43	–	–	–
	MGMT promoter unmethylation	29	12.20	4.71–19.69	0.725
	MGMT promoter methylation	10	11.63	0.00–30.22	

GTR, gross total resection; STR, subtotal resection; PR, partial resection; OS, overall survival; RT, radiotherapy; TMZ, temozolomide; MGMT, O-6-methylguanine DNA methyltransferase; KPS, Karnofsky performance status. HR, hazard ratio; CI, confidence interval. *Statistical significance.

No specimen demonstrated a *IDH1* or *IDH2* mutation according to Sanger sequencing. MGMT expression was negative in 18 of 35 cases (51.43%) by IHC. 10 of 43 cases confirmed MGMT promoter methylation by PCR (23.26%).

### Clinical and Molecular Associations With Survival Outcome

The impact of various clinical factors such as age, sex, molecular parameters on overall survival (OS) was analyzed and presented using the log-rank test and Cox regression analysis (**Table 3**). Of note, some patients could not complete their serial post-operative MRIs due to poor baseline and postoperative KPS, resulting in censored data for the assessment of progression-free survival (PFS). Therefore, we focused on OS and the relationship between the clinical and molecular factors.

The median OS was 12.83 months for the whole cohort, and the 12-, 24-month OS rates were 53.8% and 40.2%, respectively. According to both univariate and multivariate analyses, both better preoperative KPS (≥70) (*p* = 0.017, 95% CI: 0.11-10.32, HR = 0.29) and treatment with adjuvant radiotherapy (*p* < 0.001, 95% CI: 0.02–0.23, HR = 0.07) were significantly associated with a better OS prognosis. Age, sex, location of the tumor and the extent of surgical resection failed to show statistical significance upon univariate analysis.

On assessment of the patients’ molecular markers, status of MGMT promoter methylation and H3K27me3 status did not influence prognosis in our cohort. P53 overexpression demonstrated a worse prognosis by univariate analysis (*p* = 0.002). Using a multivariate model, P53 retention remained as a significantly favorable predictor of longer OS (*p* = 0.008, 95% CI: 0.08–0.68, HR = 0.23). Loss of ATRX failed to demonstrate a significant impact on OS (*p* = 0.17, 95% CI: 5.89–18.51) by univariate analysis. However, it was determined as an independent factor associated with the poor prognosis by multivariate analysis (*p* = 0.021, 95% CI: 0.01–0.69, HR = 0.09).

**Figure 2:**
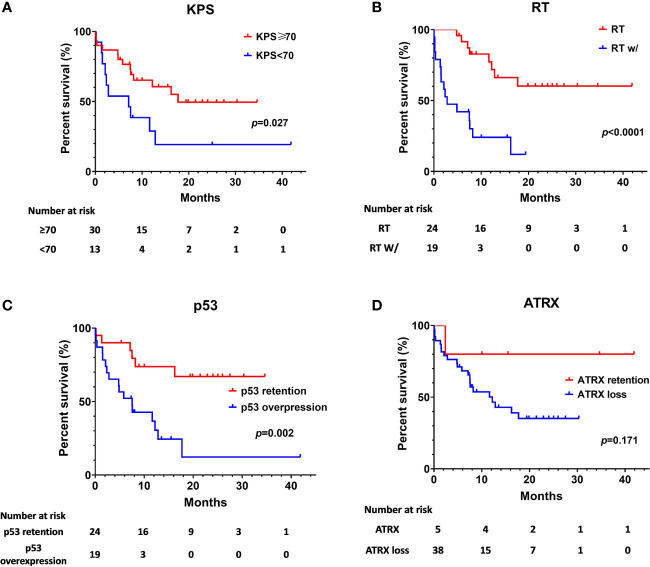
Kaplan-Meier estimates of overall survival stratified by Preoperative KPS **(A)**, radiotherapy **(B)**, p53 status **(C)**, and ATRX status **(D)**.

**Figure 3:**
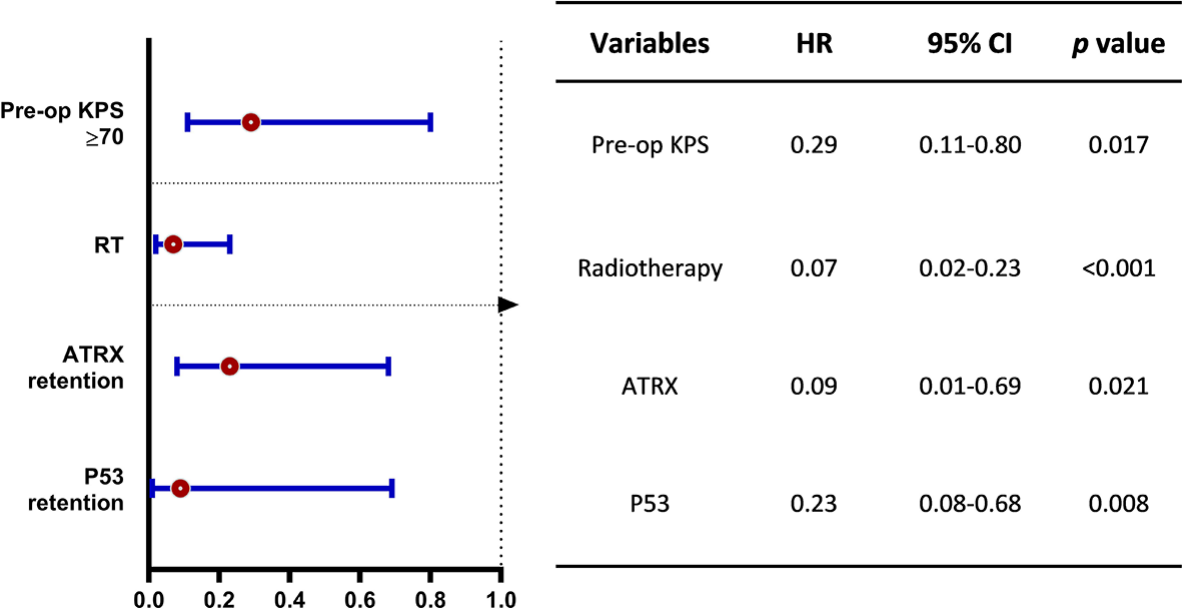
Multivariate analysis on overall survival benefit (OS). The forest plot shows the benefit of OS stratified by better preoperative KPS (≥70), treatment of adjuvant radiotherapy and p53 retention for DMG patients with H3K27M mutation. HR, hazard ratio; CI, confidence interval.

**Figure 4:**
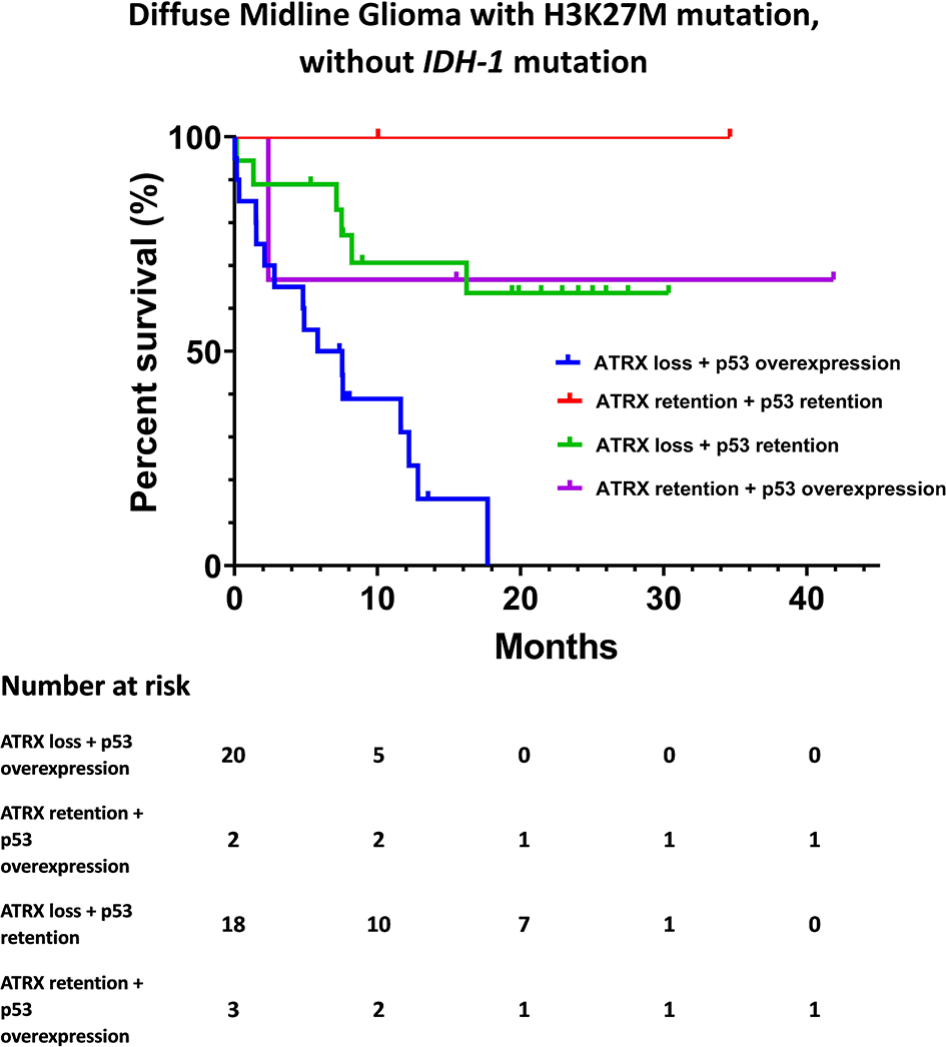
Kaplan-Meier survival analysis by ATRX status and p53 status for DMG cases without *IDH-1* mutation.

The Kaplan-Meier survival analysis of OS, stratified by KPS, adjuvant radiotherapy, ATRX, and P53, respectively, are shown in **Figure 2**. Multivariate analyses are presented in **Figure 3**. In particular, we noticed the cooperative alterations for p53 and ATRX. Both ATRX loss and p53 overexpression was observed in 20 cases (46.51%) in the cohort, which also implied a worse survival according to Kaplan-Meier analysis (**Figure 4**).

Adult and pediatric cases were also stratified and analyzed (**Supplementary Figure 2** and **Supplementary Table 2**). Based on our current data, there was no significant difference in OS between adult and pediatric groups (*p* = 0.282).

Representative IHC images are shown in **Supplementary Figure 3**.

## Discussion

As the knowledge of molecular markers in the context of malignancy increases, our understanding of gliomas has dramatically increased. Molecular pathology has become of utmost importance in the diagnosis and treatment of various CNS tumor subtypes. Diffuse midline glioma with H3K27M mutation is a novel entity defined by the 2016 WHO classification system (1, 4). Evidence has suggested that tumors with a H3K27M mutation are associated with dismal survival compared with H3K27M wildtype tumors (2, 11). As a distinct entity of glioma, DMG with H3K27M mutation typically occurs in children and corresponds to a grade IV lesion (6, 9). To date, analysis of DMG with H3K27M mutation has been mainly limited to the pediatric population. Our present study uniquely explores the clinical and molecular characteristics of 43, mostly adult, cases of diffuse midline glioma with H3K27M mutations treated as a large, tertiary medical center in China. Based on our results, a higher preoperative KPS, use of adjuvant radiotherapy, and P53 retention are found to be individual positive prognostic factors for better overall survival in this unique patient cohort.

### The Clinical Features of DMG With H3K27M Mutation

Since the revision of WHO 2016 classification, H3K27M status was introduced as a routine test at our institution in January 2017. According to our EMR, the number of diagnosed cases of DMG with H3K27M mutation only accounts for 4.69% for glioma patients. The median age of our cohort is 38 years old, which is in line with previous reports indicating a more frequent occurrence in children and young adults (2). Although males make up the majority of cases, sex is not associated with prognosis. The lesion most commonly invaded thalamus and midbrain in our cohort, which is consistent with previous reports of a thalamic predominance of DMGs in adults (9). Likewise, thalamic-related symptoms are most commonly observed, which are the manifestations of damage to the vital structures, and may result to motor function deficits (12). Preoperative KPS is closely related to tumor location and severity of the patients’ baseline clinical function. In line with prior studies on glioma, our results suggest that a poor pre-op KPS is significantly associated with poor prognosis.

Extent of resection is typically a strong predictor of survival prognosis for most gliomas, especially the high grade gliomas, such as glioblastoma (12, 13). However, since DMGs are deep-seated lesions that are located in eloquent CNS regions, the ability to achieve a safe maximal resection is substantially limited. As a result, the historical diagnosis of DMG has been traditionally made by radiographic assessment alone. With the advancement of intraoperative technologies, safe, maximal resection and/or biopsy is now possible for this unique and challenging patient population. In our study, PR and STR are preferred for most lesions, with GTR limited to specific lesions. For example, brainstem gliomas are generally treated with biopsy or surgical debulking. This surgical strategy was adopted according to the high potential risk of surgical morbidity and the dismal clinical behavior of tumors located within the midline structures that carry a H3K27M mutation (14, 15). Evidence has indicated that a greater extent of tumor resection did not provide a prognostic benefit for DMG with H3K27M mutation (2). Our current research does not find strong relevance between the extent of resection and OS for DMG cases as well. Thus, we do not consider that a more aggressive surgical intervention should be attempted for these cases. However, given the limited number of patients in this study, future studies are needed with a larger patient cohort in order to substantiate or dispute this experience.

Although adjuvant chemoradiation is typically advised after surgery, only 24 patients (RT only and RT+TMZ) in our present series received RT. Given the limited treatment options for DMG, our limited data suggests that patients may have a survival benefit with adjuvant RT (**Figure 2**). Various factors might impact the choices for the adjuvant therapy, such as socioeconomic background, education and personal expectation (15). In our cohort, we find that low postoperative KPS, lower family psychological expectation and socioeconomic consideration are main reasons behind the high RT drop-out. Some patients do not have chance to get radiotherapy due to severe postoperative complications and low KPS, such as Case No. 16 and No. 27. After surgical intervention and formal pathological diagnosis, family members tend to have much lower psychological expectation, which may lead them to be less active in following treatments. This factor may also lead to radiotherapy absence. In addition, although the majority of the patients are covered by National Health Care, and around 2/3 of a standard radiotherapy bill could be reimbursed in China, there are still patients and families who are unable to afford or continue further treatment, such as RT or TMZ. Ultimately, the present study implies that a substantial room for improvement exists for the optimal management of DMGs in China.

### Molecular Features of DMG With H3K27M Mutation

Histone gene mutations in H3F3A or HIST1H3B/C, encoding for histone H3.3 and H3.1 respectively, are considered an overall dismal prognostic indicator for patients with a DMG (3, 10). TP53 is a pivotal tumor suppressor gene and *TP53* mutation has a significant overlap with H3K27M mutations according to previous studies (16) and is in line with our study. For diffuse intrinsic pontine gliomas (DIPG) with H3K27M mutation, about 60–80% of *TP53* mutations have been associated with an aggressive course (5). Similar results were seen in our cohort andp53 overexpression was found to be associated with worse survival prognosis for both pediatric and adult patients (**Supplementary Table 2** and **Supplementary Figure 2**).

For pediatric patients, the mutation and loss of ATRX expression occur in about 1/3 of specimens and is even more common in DIPG (15). Our series found an exceptionally higher (38/43, 88.37%) incidence of ATRX loss in both pediatric (10/13) and adult (28/30) patients, which is prevalent in the thalamus and brainstem. ATRX loss was found to be an independent predictor of poor prognosis in our series when included in the multivariate model. Based on this finding, future larger studies are warranted.

Interestingly, evidence suggests that there are cooperative genetic alterations for DMG with H3K27M mutation, particularly in relation to *TP53* and *ATRX* mutations without *IDH-1* mutation (17). ATRX loss and p53 overexpression in our cohort was associated with a dismal survival expectation (**Figure 4**). However, these results should be interpreted with extreme caution given the limited number of cases included in the present study.

H3K27me3 functions as transcriptional repressor and is closely related to posttranslational modifications (18). Previous researches have shown that reduced H3K27me3 levels lead to extensive transcriptional modification of tumor cell regulation, and has been associated with an unfavorable prognosis in several cancers (19). The expression of H3K27me3 (13/19, 68.42%) is decreased in most H3K27M mutant cases. Although not statistically significant in our cohort, evidence suggests that the cooperative alteration of K27M–mediated loss of H3K27me3 is context-independent (20).

MGMT promoter methylation is comparatively rare for DMG patients (21). In our present study, the IHC results suggest a high rate of MGMT negative expression (18/35 cases). Meanwhile, qMSP confirms that MGMT methylation is not rare (10/43) in our cohort. In addition, we noticed a discrepancy regarding the MGMT status between IHC and qMSP. Evidence has suggested that the predictive power of IHC for OS appears weaker than other methylation assays (22) and the clinical utility of MGMT IHC is still controversial. Thereby, we adopted qMSP results for the analysis.

DMG with H3K27M mutation is still a fatal disease with dismal prognosis. Recently epigenetic studies have greatly broadened our understanding of its biological behavior and underlying mechanism. CSF analysis has also shown promise for the diagnosis and monitoring treatment response (23). In order to better understand and optimize survival in this unique and challenging patient population, further study is needed.

### Study Limitations

Several limitations inherent to the retrospective nature of our present study must be carefully considered. First, as a rare entity in glioma, the incidence of DMG with H3K27M mutation is substantially low. The small number of patients found for our cohort implies that the statistical power is limited in analyzing the relevant clinical and molecular factors. Second, IHC staining was utilized for most analyses in our study, and sequencing was only partially applied. Therefore, we could not further evaluate more detailed histone mutations, such as H3.1 and H3.3 mutations, which limited us to explore other genetic biomarkers and potential therapeutic effects.

## Conclusions

Despite these limitations, we retrospectively summarized the clinical and molecular features of 43 patients diagnosed with a diffuse midline glioma with H3K27M mutation over a three-year period at our institution. DMG with H3K27M mutation is associated with poor survival, with a median OS of 12.83 months in our study. Poor preoperative KPS, absence of adjuvant radiotherapy, and P53 overexpression were found to be independent prognostic factors related to worse overall survival. In addition, cooperative genetic alterations, such as loss of ATRX and p53 overexpression without *IDH-1* mutation, might help us to better understand the pathogenesis and appropriate treatment strategies for gliomas with H3K27M mutation.

## Data Availability Statement

The raw data supporting the conclusions of this article will be made available by the authors, without undue reservation.

## Ethics Statement

Ethical review and approval was not required for the study on human participants in accordance with the local legislation and institutional requirements. Written informed consent from the participants’ legal guardian/next of kin was not required to participate in this study in accordance with the national legislation and the institutional requirements.

## Author Contributions

YuanW and L-LF wrote the main manuscript text. YuanW, LG, and LW designed the study. YuanW, L-LF, P-GJ, J-HL, YueW, Y-RX, and ES helped to conduct the study, collected and analyzed data. S-CG, Y-LZ, NW, ML, MX, and MC provide the service and technical support for this study. G-DG and YQ supervised this study. LW and YuanW prepared tables. All authors reviewed the manuscript. All authors contributed to the article and approved the submitted version.

## Funding

This work was supported by National Natural Science Foundation of China (81601100 and 81772661). 

## Conflict of Interest

The authors declare that the research was conducted in the absence of any commercial or financial relationships that could be construed as a potential conflict of interest.

## Supplementary Material

Supplementary Figure 1 Representative images of extent of tumor resection (EOR). **(A)** Gross total resection (GTR) was defined as no residual tumor remnant (100%) with postoperative MRI images (A5-A8) comparing to preoperative MRI images (A1-A4). **(B)** Subtotal resection (STR) was defined as less than 90% extent of resection (preoperative MRI images B1-B4; postoperative MRI images B5-B8). **(C)** Partial resection (PR) was defined as less than 50% resection of the tumor (preoperative MRI images C1-C4; postoperative MRI images C5-C8).

Supplementary Figure 2 Kaplan-Meier survival analysis stratified by adults (n = 30) and children (n = 13).

Supplementary Figure 3 Diffuse middle glioma show variable histology features. The tumor cells can be small and monomorphic that is the same as diffuse astrocytoma with WHO grade II **(A)**. But also can show pleomorphic with necrosis and microvascular proliferation like glioblastoma **(B)**. Both of them express H3K27M **(C** and **D)**. Some cases show p53 immunonegative **(E)**. Others overexpress p53 protein **(F)**, suggesting an underlying TP53 mutation.
