# Supplementary figures and images for: Clinical Features and Molecular Markers on Diffuse Midline Gliomas With H3K27M Mutations: A 43 Cases Retrospective Cohort Study
Source: Front Oncol. 2021 Feb 15;10:602553. doi: 10.3389/fonc.2020.602553 (PMC7917281; doi:10.3389/fonc.2020.602553)

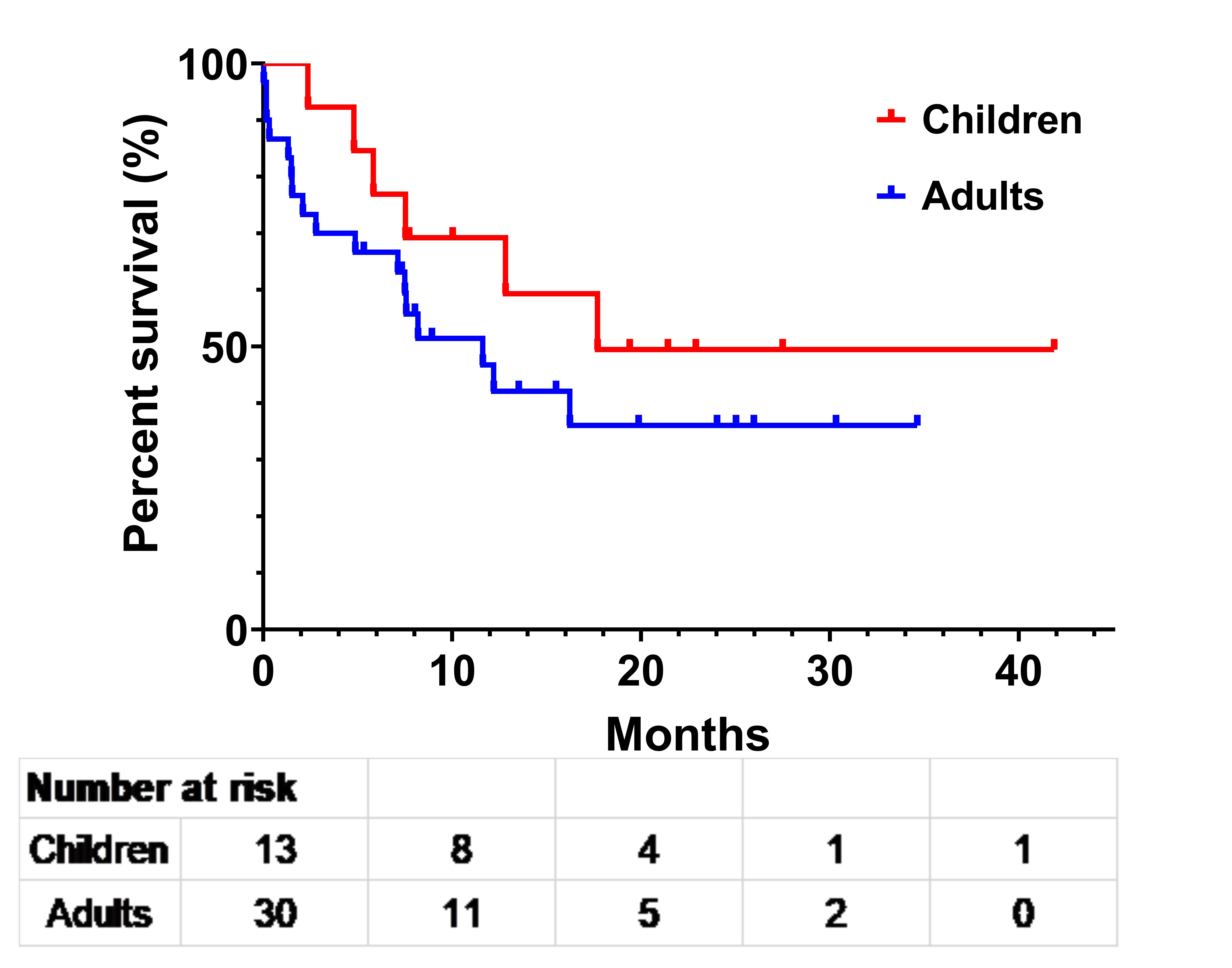

Supplement: Supplementary Figure 1 — Representative images of extent of tumor resection (EOR). (A) Gross total resection (GTR) was defined as no residual tumor remnant (100%) with postoperative MRI images (A5-A8) comparing to preoperative MRI images (A1-A4). (B) Subtotal resection (STR) was defined as less than 90% extent of resection (preoperative MRI images B1-B4; postoperative MRI images B5-B8). (C) Partial resection (PR) was defined as less than 50% resection of the tumor (preoperative MRI images C1-C4; postoperative MRI images C5-C8). [file Image_1.jpeg]

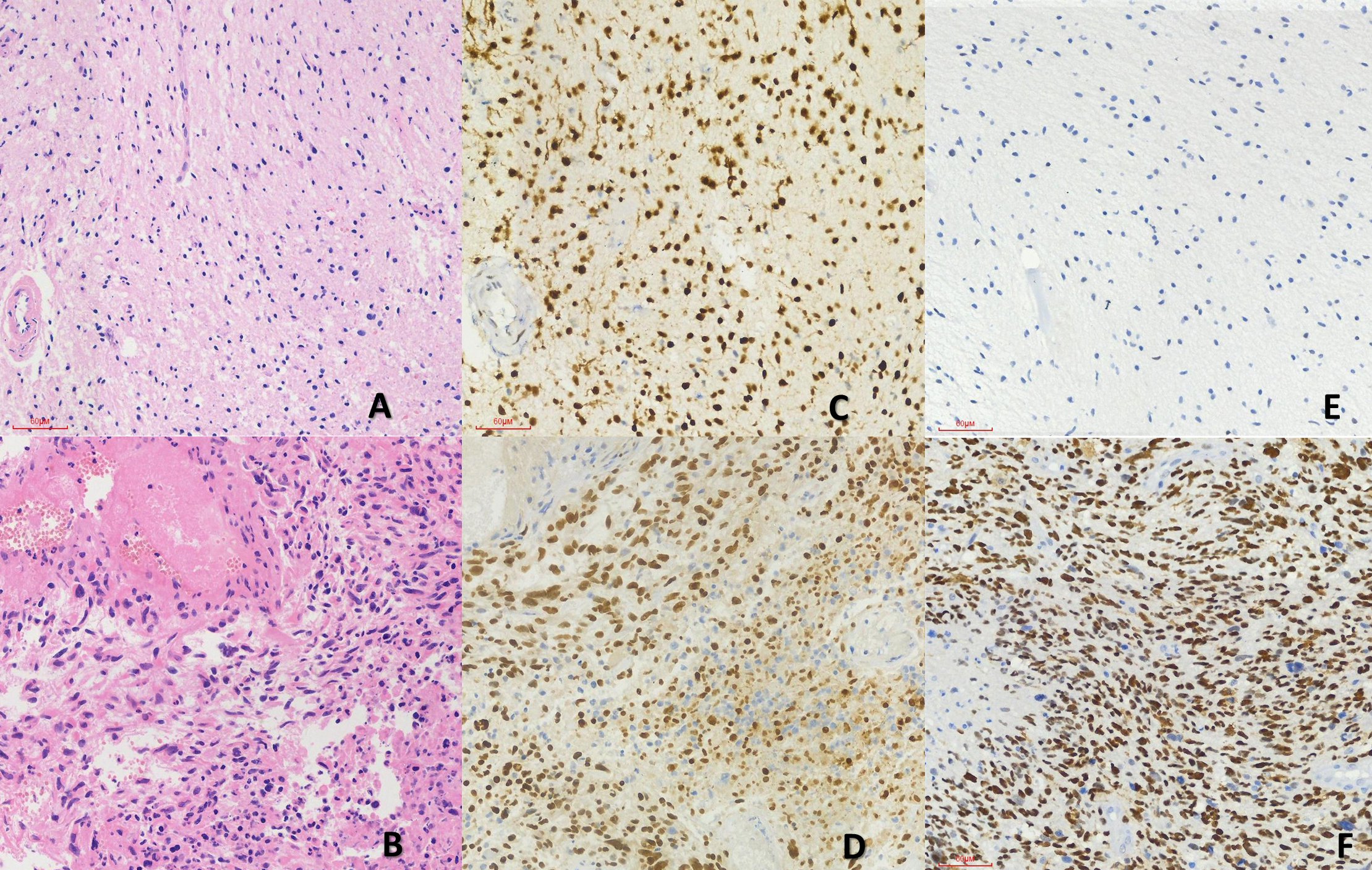

Supplement: Supplementary Figure 2 — Kaplan-Meier survival analysis stratified by adults (n = 30) and children (n = 13). [file Image_2.jpeg]

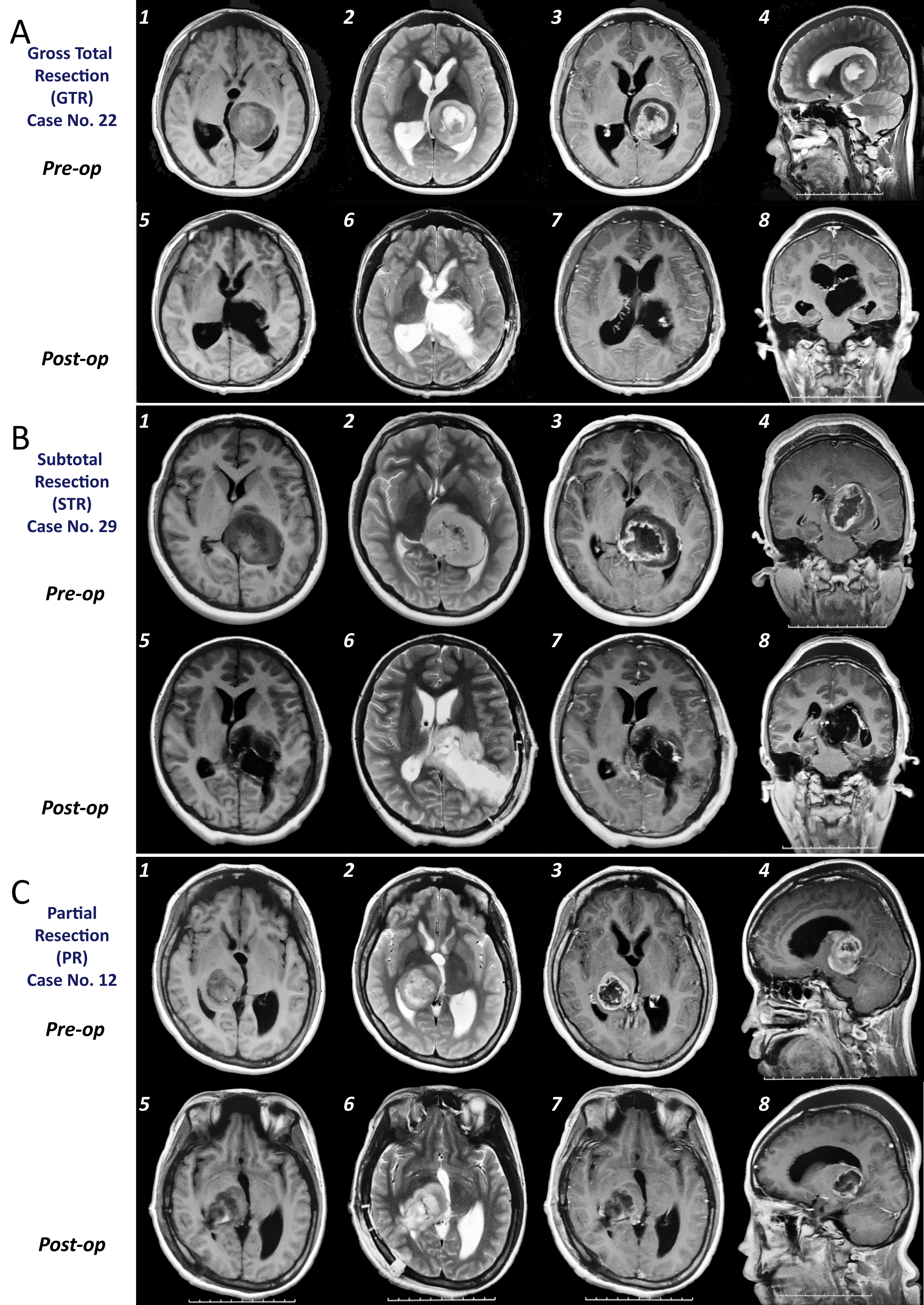

Supplement: Supplementary Figure 3 — Diffuse middle glioma show variable histology features. The tumor cells can be small and monomorphic that is the same as diffuse astrocytoma with WHO grade II (A). But also can show pleomorphic with necrosis and microvascular proliferation like glioblastoma (B). Both of them express H3K27M (C and D). Some cases show p53 immunonegative (E). Others overexpress p53 protein (F), suggesting an underlying TP53 mutation. [file Image_3.jpeg]
