# Supplementary material for: Clinical Features and Molecular Markers on Diffuse Midline Gliomas With H3K27M Mutations: A 43 Cases Retrospective Cohort Study
Source: Front Oncol. 2021 Feb 15;10:602553. doi: 10.3389/fonc.2020.602553 (PMC7917281; doi:10.3389/fonc.2020.602553)
Supplement: Supplementary file 4 [file Table_1.docx]

**Supplementary Table 1. Status of the selected molecular markers**

| **Case ID** | **IDH1** | **MGMT Protein expression (IHC)** | **MGMT promoter status (qMSP)** | **ATRX** | **P53** | **Ki67 %** | **H3K27Me3** |
| --- | --- | --- | --- | --- | --- | --- | --- |
| 1 | WT | (-) | Unmet | Retention | Overexpression | 15 |  |
| 2 | WT | Null | Unmet | Loss | Overexpression | 40 |  |
| 3 | WT | Null | Met | Loss | Retention | 40 |  |
| 4 | WT | (-) | Unmet | Loss | Overexpression | 50 |  |
| 5 | WT | (+) | Unmet | Loss | Retention | 5 |  |
| 6 | WT | (-) | Unmet | Loss | Retention | 60 |  |
| 7 | WT | (-) | Met | Loss | Overexpression | 40 |  |
| 8 | WT | (+) | Unmet | Loss | Retention | 3 |  |
| 9 | WT | (+) | Unmet | Loss | Overexpression | 40 | Expression |
| 10 | WT | Null | Null | Loss | Retention | 5 |  |
| 11 | WT | (+) | Met | Retention | Overexpression | 10 | Expression |
| 12 | WT | (+) | Unmet | Retention | Overexpression | 30 | Decreased |
| 13 | WT | (+) | Unmet | Retention | Overexpression | 40 | Decreased |
| 14 | WT | (-) | Unmet | Retention | Retention | 60 | Decreased |
| 15 | WT | (+) | Unmet | Loss | Overexpression | 8 | Decreased |
| 16 | WT | (-) | Met | Loss | Overexpression | 10 | Decreased |
| 17 | WT | (+) | Unmet | Loss | Retention | 2 | Decreased |
| 18 | WT | (+) | Unmet | Retention | Overexpression | 10 | Decreased |
| 19 | WT | (-) | Met | Loss | Overexpression | 30 | Decreased |
| 20 | WT | Null | Null | Loss | Retention | 8 | Decreased |
| 21 | WT | (+) | Unmet | Loss | Overexpression | 60 | Expression |
| 22 | WT | (+) | Unmet | Retention | Retention | 3 | Decreased |
| 23 | WT | Null | Unmet | Loss | Overexpression | 70 | Expression |
| 24 | WT | (+) | Unmet | Retention | Overexpression | 40 | Expression |
| 25 | WT | Null | Unmet | Loss | Retention |  | Decreased |
| 26 | WT | (+) | Unmet | Loss | Retention | 40 | Expression |
| 27 | WT | (+) | Met | Loss | Overexpression | 30 |  |
| 28 | WT | (+) | Unmet | Loss | Overexpression | 20 |  |
| 29 | WT | (+) | Met | Loss | Overexpression | 10 |  |
| 30 | WT | (+) | Unmet | Loss | Overexpression | 10 |  |
| 31 | WT | (-) | Unmet | Loss | Retention | 10 |  |
| 32 | WT | (+) | Unmet | Loss | Overexpression | 40 |  |
| 33 | WT | (-) | Unmet | Retention | Overexpression | 60 |  |
| 34 | WT | Null | Null | Loss | Retention | 20 |  |
| 35 | WT | (-) | Unmet | Loss | Retention | 5 |  |
| 36 | WT | (-) | Unmet | Loss | Retention | 5 |  |
| 37 | WT | (-) | Met | Loss | Retention | 3 |  |
| 38 | WT | (-) | Unmet | Loss | Overexpression | 30 |  |
| 39 | WT | Null | Null | Retention | Retention | 1 |  |
| 40 | WT | (-) | Unmet | Loss | Retention | 1 |  |
| 41 | WT | (+) | Met | Loss | Retention | 3 |  |
| 42 | WT | (-) | Met | Loss | Overexpression | 35 | Decreased |
| 43 | WT | (+) | Unmet | Loss | Retention | 5 | Decreased |

*IDH* WT, *IDH* wild-type; MGMT, O-6-methylguanine DNA methyltransferase; Met, methylation; Unmet, unmethylation; (-), MGMT negative expression; (+), MGMT positive expression.

IHC, immunohistochemistry; aMSP, methylation-specific quantitative PCR.
