# Supplementary material for: Clinical Features and Molecular Markers on Diffuse Midline Gliomas With H3K27M Mutations: A 43 Cases Retrospective Cohort Study
Source: Front Oncol. 2021 Feb 15;10:602553. doi: 10.3389/fonc.2020.602553 (PMC7917281; doi:10.3389/fonc.2020.602553)
Supplement: Supplementary file 5 [file Table_2.docx]

**Supplementary Table 2: Univariate analysis on overall survival in pediatric and adult patients of diffused midline glioma with H3K27M mutant**

| **Variables** | **Cohort** | **Children** | | | | **Adults** | | | |
| --- | --- | --- | --- | --- | --- | --- | --- | --- | --- |
|  |  | ***n*** | **Median/**  **mean OS (months)** | **95% CI** | ***p* value** | ***n*** | **Median/**  **mean OS**  **(months)** | **95% CI** | ***p* value** |
| Sex | Male | 6 | 17.7 | 0.00-37.01 | 0.531 | 20 | 12.20 | 4.36-20.04 | 0.873 |
|  | Female | 7 | 19.46 | 12.42-26.49 |  | 10 | 8.20 | 1.23-15.17 |  |
| Extent of resection | GTR or STR | 6 | 13.62 | 6.14-21.09 | 0.472 | 14  1166 | 16.23 | 10.89-25.33 | 0.296 |
|  | PR or Biopsy | 7 | 27.84 | 16.23-39.46 |  | 16 | 7.57 | 6.29-8.85 |  |
| Pre-op KPS | < 70 | 4 | 7.53 | 0.00-17.78 | 0.187 | 9 | 2.80 | 0.76-4.85 | 0.068 |
|  | ≥ 70 | 9 | 21.05 | 14.93-27.16 |  | 21 | 16.23 | 3.27-29.19 |  |
| Adjuvant therapy | RT | 11 | 26.57 | 16.69-36.46 | 0.353 | 13  17 | 27.71 | 21.05-34.34 | <0.001* |
|  | RT without | 2 | 6.20 | 0.89-11.51 |  | 17 | 2.80 | 5.18-17.08 |  |
| Molecular markers | H3K27Me3 retention retention | 2 |  | - | 0.090 | 4 | - | - | 0.049* |
|  | H3K27Me3 decreased | 2 | - | - |  | 11 | - | - |  |
|  | ATRX retention | 3 | 28.70 | 7.63-49.78 | 0.925 | 2 | - | - | 0.150 |
|  | ATRX loss | 10 | 17.70 | 12.25-24.17 |  | 28 | - | - |  |
|  | P53 retention | 5 | - | - | 0.013* | 15 | 22.36 | 14.84-29.87 | 0.025* |
|  | P53 overexpression | 8 | - | - |  | 15 | 4.87 | 0.00-11.41 |  |
|  | *IDH1* (WT) | 13 | - | - | - | 30 | - | - | - |
|  | *IDH2* (WT) | 13 | - | - | - | 30 | - | - | - |
|  | MGMT prompter unmethylation | 10 | 23.69 | 12.46-34.92 | 0.539 | 19 | 12.2 | 4.30-20.11 | 0.360 |
|  | MGMT prompter methylation | 3 | 18.55 | 17.37-19.73 |  | 7 | 1.50 | 1.06-1.94 |  |

GTR, gross total resection; STR, subtotal resection; PR, partial resection; OS, overall survival; RT, radiotherapy; TMZ, temozolomide; MGMT, O-6-methylguanine DNA methyltransferase; KPS, Karnofsky performance status. HR, hazard ratio; CI, confidence interval. *Statistical significance.
